# Magnetic resonance diffusion tensor imaging in psychiatry: a narrative review of its potential role in diagnosis

**DOI:** 10.1007/s43440-020-00177-0

**Published:** 2020-10-30

**Authors:** Piotr Podwalski, Krzysztof Szczygieł, Ernest Tyburski, Leszek Sagan, Błażej Misiak, Jerzy Samochowiec

**Affiliations:** 1 Department of Psychiatry, Pomeranian Medical University, 26 Broniewski, 71-457 Szczecin, Poland; 2 Institute of Psychology, SWPS University of Social Sciences and Humanities, Poznań, Poland; 3 Department of Neurosurgery, Pomeranian Medical University, Szczecin, Poland; 4 Department of Genetics, Wroclaw Medical University, Wroclaw, Poland

**Keywords:** Diffusion tensor imaging, DTI, Fractional anisotropy, Schizophrenia, Affective disorders, Personality disorders

## Abstract

Diffusion tensor imaging (DTI) is an imaging technique that uses magnetic resonance. It measures the diffusion of water molecules in tissues, which can occur either without restriction (i.e., in an isotropic manner) or limited by some obstacles, such as cell membranes (i.e., in an anisotropic manner). Diffusion is most often measured in terms of, inter alia, fractional anisotropy (FA), mean diffusivity (MD), radial diffusivity (RD), and axial diffusivity (AD). DTI allows us to reconstruct, visualize, and evaluate certain qualities of white matter. To date, many studies have sought to associate various changes in the distribution of diffusion within the brain with mental diseases and disorders. A better understanding of white matter integrity disorders can help us recognize the causes of diseases, as well as help create objective methods of psychiatric diagnosis, identify biomarkers of mental illness, and improve pharmacotherapy. The aim of this work is to present the characteristics of DTI as well as current research on its use in schizophrenia, affective disorders, and other mental disorders.

## Introduction

Despite their diversity in terms of psychopathological symptoms and causes (etiopathogenesis), many mental illnesses and disorders are characterized by certain common features in their clinical pictures. One of the most important and frequently studied etiopathogenetic factors are disorders of the brain’s structure and function. Knowledge about the relationships of structural and functional anomalies—in both gray matter and white matter—with psychopathological symptoms is still incomplete [1]. The study of such relationships is important for the development of psychiatry and for better understanding the neurobiological bases of mental diseases and disorders. Finding objective psychiatric diagnostic methods has been a goal for clinicians since the nineteenth century. This began with Freud attempting to distinguish between hysterical paralysis, sensory disorders, pseudo-seizures, amnesia, and neurological conditions; it has proven a difficult task for clinicians. In the 1970s, with the progress of technology, doctors were first given the opportunity to do brain imaging in vivo [2].

Magnetic resonance imaging (MRI) allows imaging of the brain with very high resolution. Moreover, MRI enables us to study not only the structure but also the function of the brain by measuring the flow of oxygenated blood with blood-oxygen-level-dependent (BOLD) imaging. Researchers in the field of neuroscience have begun a discussion on the currently used classifications and diagnostic criteria for mental illnesses, which are still based on subjective clinical assessment [3]. Neuroimaging studies have identified structural and functional disturbances of the brain that can be associated with mental disorders. However, they do not always directly correspond with the criteria of current diagnostic classifications. Psychiatric diagnosis remains mainly based on descriptions of the patient's behavior. As a result, neurobiological causes of mental illnesses and disorders are not fully taken into consideration. This is why, biomarkers are of great importance for preclinical research and everyday clinical practice. They can be used to diagnose as well as to assess the severity of a disease, its prognosis, and its expected response to the applied therapeutic action. Hence, the identification of neuroimaging biomarkers is necessary to create an objective method of diagnosis [1, 4].

There has been intense development in the field of imaging in recent decades. Neuroimaging is widely employed in psychiatry, as it can be used not only as a supplementary method for making a diagnosis, but also in the search for new drugs, to study the mechanisms that organize the mental life of the individual, and to identify the causes of neurobiological mental diseases. Neuroimaging can be helpful in assessing the effectiveness of treatment, as well. This is a complicated problem, for which various types of psychopathological scales are currently being employed. The use of imaging to measure therapeutic effects can increase the objectivity of this process [4]. Unlike neurological diseases, mental illnesses are not characterized by the presence of neurological symptoms that can be quickly and precisely recognized and assessed. As with neuroradiological diagnostics, neuroimaging may not show subtle changes that can be assessed by routine observation [5].

The number of available neuroimaging techniques has increased in recent decades. The use of functional magnetic resonance imaging (fMRI), positron emission tomography (PET), and single-photon emission computed tomography (SPECT) has enriched the arsenal of psychiatry [4]. One of the most interesting techniques that can be applied in the study of people with mental illness is diffusion tensor imaging (DTI).

The aim of this work is to present the DTI neuroimaging method and discuss its use in psychiatry. It will be illustrated using schizophrenia, affective disorders, and other mental disorders as examples. The limitations of this method will also be presented.

## Characteristics of diffusion tensor imaging

DTI uses magnetic resonance imaging to assess the diffusion properties of water in intra- and extra-cellular space. It does not require the use of additional equipment, contrasting substances, or chemical markers [6]. Diffusion imaging techniques have been used for about 30 years, and enable non-invasive imaging and examination of the structure of various tissues. In contrast to classical magnetic resonance imaging techniques, DTI allows the indirect assessment of tissue microstructure.

The movement of water in tissue is determined by the type of tissue, its architecture, integrity, and the presence of barriers [6]. Based on these features, it can be used to study the properties of white matter fibers throughout the whole brain. DTI uses the properties of water diffusion in various closed spaces. Due to their internal energy, water molecules in a liquid state undergo continual chaotic movements known as Brownian motion. In an environment without any barriers (e.g., in a glass), water molecules will disperse in a uniform manner, moving evenly in all directions. This type of diffusion is called isotropic diffusion. When water molecules encounter barriers, they stop moving evenly in all directions. This type of diffusion is called anisotropic diffusion. Anisotropic diffusion occurs in highly organized tissues; for example, in nerve fibers, water diffuses in a relatively free manner along, while in a much-limited manner across the nerve fiber. On the other hand, in gray matter, which is not as regularly ordered as white matter, isotropic diffusion will dominate. A further example would be cerebrospinal fluid, where water diffuses evenly in all directions, so the diffusion here is isotropic [7].

Thanks to magnetic resonance imaging, it is possible to measure water diffusion in a single voxel (typically a three-dimensional pixel representation) over a timespan of a few milliseconds, thereby recording the magnitude and direction of such diffusion. These data are then used to create a mathematical construct known as a “tensor”, which graphically represents diffusion within the voxel. Combining the data collected from the entire area being studied allows us to model the course of bundles and graphically represent the diffusion distribution. These data, in turn, allow us to conduct a tractography (i.e., to graphically represent the bundles). This provides a very accurate model of a tissue (e.g., white matter), which, in the past, was only possible in post-mortem studies.

DTI analysis uses several coefficients which are calculated for the corresponding diffusion tensor. The most frequently used are fractional anisotropy (FA), mean diffusivity (MD), radial diffusivity (RD), and axial diffusivity (AD). The most common indicator of all is FA. This is a standardized measure with values between 0 and 1, and it roughly shows how much water within the micro- and macro-structure undergoes anisotropic diffusion. It is often interpreted as an indicator of white matter integrity (a high FA value indicates high white matter integrity), as it is associated with the level of axonal ordering within bundles, their myelination, and any disruptions they might have sustained [8]. Reduction of anisotropy may be an indicator of impaired integrity of white matter; however, there may be various pathophysiological phenomena underlying these changes that have not been fully recognized yet. MD represents the overall limits of diffusion, regardless of its direction. The lower the MD, the more restricted the structure. AD and RD describe the extent of diffusion in planes parallel and perpendicular, respectively, to the main axis of the tested tensor. An increase of AD indicates increased ordering in a given structure, while an increase in RD is associated with a decrease in ordering [7]. The proposed interpretation of selected indicators is presented in Table 1.

**Table 1:** Diffusion measures commonly used in the interpretation of DTI data in the brain [7, 9–11]

Factors	Fractional anisotropy (FA)	Mean diffusivity (MD)	Radial diffusivity (RD)	Axial diffusivity (AD)
Demyelination	↓	↑	↑	
Inflammation	↓	↑		
Edema	↓	↑		
Tumor	↑	↓		
Necrosis	↓	↑		
Gray matter	↓	↑	↓	↑
White matter	↑	↓	↑	↓
Cerebro-spinal fluid	↓	↑	↑	↑
Ischemic stroke:				
Acute	↑	↓		
Normalization		↑		
Chronic	↓	↑		

(↑) = high value, (↓) = low value

It is difficult to use this method in everyday clinical practice due to its complexity. Conducting neuroimaging using DTI requires special data acquisition protocols, data developed to assess artifacts in the obtained data, and quality control, visualization, and subsequent mathematical analysis of the obtained data [6]. DTI results should be interpreted carefully, as they can often be ambiguous. The results of these analyses may be affected by the specific nature of the obtained data. For example, the presence of fibers oriented in different planes within a voxel can decrease the average FA value, but this decrease is not due to changes in myelin sheaths or the axon structure itself—this is known as “fiber crossing”. Knowing the limitations of the method is crucial for proper planning a study and creating an appropriate model that enables the interpretation of the results [8].

## White matter abnormalities in schizophrenia

Schizophrenia is a severe chronic neurodevelopmental disease. It hinders the functioning of a large proportion of patients to such an extent that their life in society is significantly disturbed. It affects every sphere of mental life: thought, emotions, and behavior. The prevalence of the disease in 2016 was estimated at 0.28%. Despite this relatively low prevalence, it is a common cause of disability, especially in middle-income countries. Furthermore, it is associated with shortened life expectancy and increased mortality is observed in all age groups. Schizophrenia also has increased comorbidity with other chronic diseases, such as heart disease, diabetes, some cancers, respiratory diseases, and strokes [12].

Schizophrenia has been much studied by neuroscience. The etiopathogenesis of this disease is still not fully understood. In the nineteenth century, when it began to be diagnosed as a disease based on diagnostic criteria, there were many hypotheses about its causes. Eugen Bleuler saw the causes of this disease in the disintegration of the psyche [13]. On the other hand, Carl Wernicke suggested the disconnection hypothesis, which posits schizophrenia as being due to anatomical disorders in connections between the various parts of the brain [14]. These early concepts highlighted the importance of connection pathology on the anatomical and functional levels. Wernicke’s hypothesis was brought back to life at the end of the twentieth century when, in 1988, Volkow et al. published a paper which suggested the existence of abnormalities in patterns of interactions between brain areas in schizophrenia. They based this on the observation of abnormal glucose metabolism in various areas of the brain in PET scans [15]. MRI studies on patients suffering from schizophrenia observed a general reduction in cerebral volume (first in the temporal lobe, and then in the frontal and parietal lobes), increased ventricular space, and decreased white matter volume [16].

Thanks to development in various fields of science, it has become possible to test the disconnection hypothesis. It assumes that psychopathological symptoms are caused by incorrect communication between functional regions of the brain, rather than by the disorder of one specific area of the brain. Histopathological examinations seem to confirm this, as reduced numbers of neuroglia cells, disorders of the myelin sheath structure, mitochondrial degeneration, reduction of presynaptic follicles, and neuronal atrophy have been found in post-mortem studies of people suffering from schizophrenia [17].

Many reports indicate the crucial role of the prefrontal cortex (PFC), the temporal cortex, and their connecting fibers in the etiology of schizophrenia. The PFC has broad connections with almost all cortical and subcortical areas, which gives it the unique position of controlling many cognitive functions. These functions include working memory, declarative memory, rule learning, planning, problem solving, recognition of new stimuli, attention control, motivation control, language control, inhibition of reactions, decision making, emotion control, and social cognition [18]. The main white matter fibers connecting the PFC to other areas of the brain are the cingulum, inferior fronto-occipital fasciculus, anterior thalamic radiation, arcuate fasciculus, fornix, and uncinate fasciculus. Structural and functional disorders within these structures may have a significant impact on the etiopathogenesis of schizophrenia [19].

Despite many years of research, the results of white matter structure studies are still inconsistent in patients with schizophrenia. Table 2 presents the bundles of white matter most frequently studied in psychiatry. The results of DTI in schizophrenia are inconclusive due to the variation between studies in terms of protocols, scanners, patient ages, disease duration, demographic differences, and treatment. Moreover, the pathophysiological processes underlying changes in white matter structure have not yet been described in detail. Many research results show a decrease in FA across the whole brain in patients with schizophrenia [20, 21]. Increases in MD and RD have also been reported [21]. It has also been suggested that changes in these values within white matter structures are related to the psychopathology of the disease [22].

**Table 2:** Descriptions of commonly used bundles and their functions in DTI studies [23–28]

Bundles	Type of fiber	Connections between brain areas	Functions
Corpus callosum	Commissural fiber	Connects identical cortical areas in both brain hemispheres	Processing input and output signals from each hemisphere; coordination of thought processes and behavior
Cingulum bundle	Association fiber	Connects the areas of the frontal, parietal, temporal lobes; connects the subcortical nuclei to the cingulate gyrus; connects various elements of the limbic system with each other	Controls executive functions; attention; emotional and motivational processes; socio-emotional processes; episodic memory; pain
Uncinate fasciculus	Association fiber	Connects the lateral parts of the orbito-frontal cortex with the anterior part of the temporal lobe	Episodic memory; language processes; socio-emotional processes
Arcuate fasciculus	Association fiber	Connects the frontal lobe to the temporal lobe	Speech comprehension; speech and language processes
Superior longitudinal fascicles	Association fiber	Connects the frontal lobe to the parietal lobe and partly to the temporal lobe	Visual-spatial functions; motor control; hearing and articulation processes; understanding the content of heard and read text; speech processes; using tools
Inferior longitudinal fasciculus	Association fiber	Connects the occipital lobe to the temporal lobe	Visual processes; emotional processes; behavior related to vision; visual cognition; visual memory

The corpus callosum is the white matter structure deep inside the cerebral fissure. It is the largest commissural fiber of the brain, expanding deep into both hemispheres, and it connects many distant areas of the brain with each other. It helps coordinate cognitive processes and behavior. It is believed that aberrations within this white matter structure play a significant role in the etiopathogenesis of schizophrenia [29]. Many studies have examined FA values in the corpus callosum in patients with schizophrenia [20, 21, 30, 31]. Decreases in FA are positively correlated with illness duration [32]. It turns out that these changes are more common within the body, rather than the genu, of the corpus callosum [29]. Increases in MD and RD have also been found in this area [21]. Structural changes in the corpus callosum may be present even in the first episode of the disease—such patients have decreased FA and increased RD, which is important for identifying biomarkers of this disease [33].

The cingulum is the most important white matter structure within the limbic system. It is under the cingulate gyrus and passes over the corpus callosum. It is responsible for the connection between the neocortical areas and the limbic system; it also connects the limbic system with the subcortical areas of gray matter. It plays an important role in controlling the expression of emotions, attention, motivation, and working memory. Disorders of these functions are often observed in schizophrenia [23]. Decreases in FA [20, 21, 30, 31, 34–36] and increases in RD [21] in the cingulum in patients with schizophrenia have been widely described in the literature. Moreover, changes within this white matter structure have been observed as early as during the first psychotic episode [37] and in patients at high risk of psychosis [23].

The superior longitudinal fasciculus (SLF) is another important white matter structure. It is divided into four parts. They connect the area of the frontal lobe and the operculum with the upper part of the parietal lobe (SLF I) and the angular gyrus (SLF II), and the supramarginal gyrus (SLF III) with the superior temporal gyrus (SLF IV). The fourth part of the superior longitudinal fasciculus is also called the arcuate fasciculus. It connects Wernicke's area (Brodmann's area 22) and Broca's area (Brodmann’s areas 44 and 45). Its activity is associated with many key functions necessary in everyday life, such as speech, using tools, thinking ahead, and empathy. It is also involved in the control of spatial and visual function, motor skills, the process of hearing, and comprehending that which one hears and reads [24, 38]. In the course of schizophrenia, decreases in FA are observed in this structure [20, 30, 31, 36]. Changes in RD and FA values are positively correlated with auditory hallucinations [38, 39]. Decreased AD in the arcuate fasciculus has been reported in healthy family members of patients suffering from schizophrenia [40].

The arcuate fasciculus belonging to the association fibers is a white matter structure which connects the lateral areas of the orbital–frontal cortex with the anterior part of the temporal lobe. It is involved in the performance of functions in the fields of memory, language, and controlling emotions. These processes are heavily disturbed in the course of schizophrenia. This may be caused by microstructure disorders of this nerve fiber. Changes in diffusion parameters in this area have been found in patients with schizophrenia. Decreases in FA are most frequently reported [20, 30, 36], but so too are increases in MD, AD, and RD [21]. Furthermore, healthy family members of people with schizophrenia have also been reported to have increased AD in this structure. These findings can potentially be used to identify biomarkers of the disease [41].

The main association fiber connecting the temporal lobe with the occipital lobe is the inferior longitudinal fasciculus. It is believed to be involved in many important brain functions because of the areas which it connects. This structure is responsible for recognizing objects and other people, it is involved in the process of seeing, and it is important when reading and comprehending text. Furthermore, it is responsible for visual memory and participates in the control of emotional processes [25]. Auditory hallucinations are one of the psychotic symptoms that occur in patients with schizophrenia. They can be caused by inferior longitudinal fasciculus integrity disorders, which may be explained by a decrease in FA found on the left side in patients with current hallucinations [42]. A general decrease in FA in patients with schizophrenia has also been reported [31, 36]. These changes may be present as far back as the first episode of the disease, which may be associated with the important role of this white matter system in the etiopathogenesis of schizophrenia [37].

Table 3 presents disturbances in diffusion parameters in various fiber bundles in schizophrenia. The results of these studies may suggest that schizophrenia is a disease characterized by global disorders of structural and functional communication between various areas of the central nervous system [19].

**Table 3:** Changes in diffusion parameters in the brain structures in schizophrenia [20, 21, 30, 31, 33–40, 43, 44]

	Bundles	Changes in diffusion parameters	Authors	Bundles	Changes in diffusion parameters	Authors
Schizophrenia	Cingulum	Reduced FA	Dong et al. [30]; Kelly et al. [20]; Koshiyama et al. [21]; Kubicki et al. [34]; Vitolo et al. [31]; Wang et al. [35]; Yang et al. [36]	Corpus callosum	Reduced FA	Dong et al. [30]; Kelly et al. [20]; Koshiyama et al. [21]; Vitolo et al. [31]; Yang et al. [36]
		Increased RD	Koshiyama et al. [21]		Increased MD and RD	Koshiyama et al. [21]
		Reduced FA in patients having their first episode of psychosis	Yao et al. [37]		Reduced FA and increased RD in patients having their first episode of psychosis	del Re et al. [33]
		Reduced FA and increased RD in patients at high risk of psychosis	Fitzsimmons et al. [23]	Superior longitudinal fasciculus	Reduced FA in patients with auditory hallucinations	Chawla et al. [38]
	Uncinate fasciculus	Reduced FA	Dong et al. [30]; Kelly et al. [20]; Yang et al. [36]		Reduced FA in the right superior longitudinal fasciculus in patients with deficit syndrome	Rowland et al. [44]
		Increased MD, AD, and RD	Koshiyama et al. [21]		Reduced FA	Dong et al. [30]; Kelly et al. [20]; Vitolo et al. [31]; Yang et al. [36]
		Increased MD in the left uncinate fasciculus in patients with deficit syndrome	Voineskos et al. [45]	Arcuate fasciculus	Reduced AD in healthy family members	Kubicki et al. [34]
		Reduced AD in healthy family members	Kubicki et al. [34]		Reduced FA	Vitolo et al. [31]
		Reduced FA in the left uncinate fasciculus in patients with deficit syndrome	Kitis et al. [43]		Reduced FA in patients with auditory hallucinations	Chawla et al. [38]; de Weijer et al. [39]
	Inferior fronto-occipital fasciculus	Reduced FA	Dong et al. [30]; Kelly et al. [20]; Vitolo et al. [31]; Yang et al. [36]		Increased MD in the right arcuate fasciculus in patients with deficit syndrome	Voineskos et al. [45]
		Reduced FA in the left inferior fronto-occipital fasciculus in patients having their first episode of psychosis	Yao et al. [37]	Whole brain–white matter	Reduced FA	Kelly et al. [20]; Koshiyama et al. [21]
		Reduced AD in healthy family members	Kubicki et al. [34]		Increased MD and RD	Koshiyama et al. [21]
	Inferior longitudinal fasciculus	Reduced FA	Vitolo et al. [31]; Yang et al. [36]	Fornix	Increased MD, AD, and RD	Koshiyama et al. [21]
		Increased MD in the right inferior longitudinal fasciculus in patients with deficit syndrome	Voineskos et al. [45]		Reduced FA	Kelly et al. [20]; Koshiyama et al. [21]; Vitolo et al. [31]; Yang et al. [36]
		Reduced FA in the left inferior longitudinal fasciculus in patients having their first episode of psychosis	Yao et al. [37]	Corona radiata	Reduced FA	Kelly et al. [20]
	Internal capsule	Reduced FA	Kelly et al. [20]; Vitolo et al. [31]		Increased AD and RD	Koshiyama et al. [21]

Some reports discuss the influence of neuroleptics on the integrity of white matter. Research suggests that atypical neuroleptics may have a neuroprotective effect. Previously untreated patients experiencing their first episode of the disease who underwent 6 weeks of therapy with amisulpride were found to have normalized diffusion indexes. These results indicate that dopaminolytics may have a remyelinating effect [46] and may have a protective effect on oligodendrocytes, which has been observed in the case of aripiprazole therapy [47, 48]. Other studies, however, have not found specific changes in the integrity of white matter as measured by DTI [49]. Drug resistance is a significant problem in the therapy of patients with schizophrenia. As many as one-third of patients may not improve after treatment with neuroleptics (excluding clozapine) [50]. It turns out that drug-resistant patients, in comparison to patients who respond well to antipsychotic treatment, have reduced FA values in many white matter pathways (e.g., the corona radiata, corpus callosum, internal capsule, superior longitudinal fasciculus, or arcuate fasciculus) [32, 51]. However, an increase in RD value has been observed in the corpus callosum, internal and external capsule, and the right inferior longitudinal fasciculus [32], which may make the use of other biological therapy methods more effective, improving the prognosis of such patients.

Deficit schizophrenia is a subtype of schizophrenia characterized by the presence of both primary symptoms and persistent negative symptoms, regardless of the stage of the disease and the treatment applied. It is quite a rare subtype of schizophrenia and its prevalence in the population of people suffering from schizophrenia is about 15% [52]. It was first described by Carpenter in 1988 [53] and involves impaired control of emotional reactivity, reduced cognitive functions, limited thought content, and reduced social activity [45]. This group of patients also differs in that they have lower levels of suspicion, dysphoria, and suicidal thoughts, and are less prone to abuse psychoactive substances [54]. The treatment of these negative symptoms is difficult and complete remission of symptoms is often impossible, which frequently leads to diminished social functioning in people with this subtype of schizophrenia [55].

In MRI studies, clear morphological markers of deficit schizophrenia have not yet been determined. In recent years, a number of studies using DTI have been conducted to assess the structure of white matter in patients with deficit syndrome. Changes in the values of diffusion indices have been described in the inferior longitudinal fasciculus on the right side [45], the right arcuate fasciculus [45], and in the left uncinate fasciculus [43, 45]. Decreased FA values in the corpus callosum, superior longitudinal fasciculus, and uncinate fasciculus have been found in untreated patients diagnosed with deficit syndrome [56].

Some research results suggest that the underlying cause of deficiency syndrome symptoms is impaired communication between different areas of the brain; however, there is still not enough evidence to allow certainty on the matter. Water diffusion disorders in white matter in the prefrontal cortex areas indicate that these areas play an important role here [57].

## Changes in white matter in affective diseases

DTI studies have also shed light on major depressive disorder (MDD) and bipolar disorder (BP).

Depression is the most common mental illness and one of the most common causes of absences from work [58]; it is characterized by persistent low mood, is recurrent, and seriously affects one’s functioning. It significantly reduces quality of life and shortens life expectancy.

The etiopathogenesis of depression remains unknown. In recent years, there has been an increase of diffusion imaging studies examining affective disorders. The results are, however, inconclusive. Some theories suggest that MDD is rooted in disorders of the cortico-subcortical network [59]. It has been found that people suffering from MDD have impaired white matter integrity in the connections between the frontal lobe and the limbic system, which are responsible for controlling and regulating behavior and emotions [60].

Research in this field thus far has noted various types of changes in diffusion parameters, which suggests disruption to the integrity of white matter pathways. The decrease in FA and increase in RD throughout the whole brain is consistent with disruption in the whole brain between cortical and subcortical areas [61]. The lesions can be located within association fibers or in commissural fibers.

In 2019, the ENIGMA consortium published a major meta-analysis which included DTI of 1,305 people diagnosed with MDD. A decrease in the FA index was observed in 16 out of the 25 white matter tracts of interest. The largest changes compared to the control group were found in the corona radiata, corpus callosum, and external capsule. Lesions were also observed in the superior fronto-occipital fasciculus, internal capsule, and fornix. These changes were more pronounced in patients with recurrent episodes, those who first experienced MDD after the age of 21, and patients who were not taking antidepressant medication during the study [61]. Other studies presenting changes in white matter in MDD are presented in Table 3.

More than 50% of patients suffering from non-psychotic depressive disorder do not achieve remission during pharmacotherapy with one antidepressant. There is also no clinical tool that can reliably plan completely effective therapy. Differences in white matter diffusion values in some brain structures may be a biomarker of disease remission after treatment [62]. A decrease in FA within the amygdala fibers may result in a worse response to treatment with selective serotonin reuptake inhibitors (SSRIs) [63]. A change in FA within the white matter of the limbic system may be a marker of disease remission. One DTI study had 62% accuracy in predicting remission in a sample of 102 patients undergoing treatment with antidepressants [64]. In a similar study conducted from 2008 to 2014, in which FA values ​​were measured within the stria terminalis and cingulum, over 80% specificity was obtained in predicting response to treatment. That study focused only on corticolimbic pathways, detecting nearly 30% of those who did not respond to treatment with high specificity [62]. Other DTI values can also be used to predict responses to pharmacotherapy. A decrease in AD within the external capsule is often associated with a weaker response to SSRIs [65].

Bipolar disorder is a chronic psychiatric disease with a variable course. The main psychopathological features of this disease are mania and depression, which occur with varying intensity and severity. The etiopathogenesis of this disease remains unknown [66].

In recent years, intensive research has attempted to assess the role of white matter in bipolar disorder. DTI studies may suggest that BP is also a disease associated with connection disorders in certain areas of the brain. It may be associated with disorders of the connections of the frontal and limbic areas, but also of the parietal and temporal cortices [67]. This is consistent with neuropathological studies, the results of which show a reduced number of oligodendrocytes in the cortical and subcortical areas [68].

Decreased FA appears mostly in the corpus callosum, cingulum, corona radiata, internal capsule, superior longitudinal fasciculus, and fronto-occipital fasciculus [21, 30, 61, 69]. The corpus callosum has also been found to have increased MD [21] and RD [69] in these patients. However, there is no consistency in this last parameter (RD), because it has also been observed to be reduced in this population [21]. Interestingly, within the fornix, which is part of the limbic system, an increase has been observed in MD and RD values [21].

DTI can also help distinguish between disease subtypes. It turns out that, compared to type II BP, the arcuate fasciculus presents a definite decrease in FA in type I BP. This may be of key importance for diagnosis, planning pharmacotherapy, and assessment of prognosis [70].

Suicide is a massive problem across the world—800,000 deaths a year are due to suicide, and there are 20 times this number of attempts [71]. People suffering from depressive episodes and bipolar disorder are at a very high risk of death by suicide. About 30% of people diagnosed with bipolar disorder and 15% of those diagnosed with MDD attempt suicide at least once in their life [72]. An objective assessment of the risk of such behavior may be of key importance for the care of this group of patients. In patients diagnosed with MDD who have attempted suicide, decreased FA has been observed in the fronto-occipital fasciculus, uncinate fasciculus, corpus callosum, internal and external capsule, corona radiata, and the thalamic radiations in comparison to those who have not attempted it. In people diagnosed with BP who have attempted suicide, decreased FA has been observed in the fronto-occipital fasciculus, uncinate fasciculus, corpus callosum, internal and external capsule, corona radiata, and the thalamic radiations in comparison to those who have not attempted suicide. The presence of similar changes within the brain may indicate a common pathomechanism in these affective disorders [73]. Table 4 presents, inter alia, disturbances in diffusion parameters in various fiber bundles in affective disorders.

**Table 4:** Changes in diffusion parameters in the brain structures in various psychiatric conditions [21, 30, 60, 61, 69, 70, 74–82]

	Bundles	Changes in diffusion parameters	Authors		Bundles	Changes in diffusion parameters	Authors
Bipolar disorder	Corpus callosum	Reduced FA	Dong et al. [30]; Magioncalda et al. [69]	Major depressive episode	Whole brain—white matter	Reduced FA and increased RD	van Velzen et al. [61]
		Increased MD	Koshiyama et al. [21]		Corpus callosum	Reduced FA	Jiang et al. [83]; Liao et al. [60]; van Velzen et al. [61]
		Increased RD	Magioncalda et al. [69]			Reduced FA in treatment-naive patients	Chen et al. [81]
		Reduced RD	Koshiyama et al. [21]		Internal capsule	Reduced FA	van Velzen et al. [61]
	Corona radiata	Reduced FA	Magioncalda et al. [69]			Reduced FA in anterior limb	Chen et al. [81]
		Increased RD	Magioncalda et al. [69]		Cingulum	Reduced FA	van Velzen et al. [61]
		Increased AD	Koshiyama et al. [21]		Superior longitudinal fasciculus	Reduced FA	Jiang et al. [83]
	Internal capsule	Reduced AD	Lan et al. [82]		Inferior longitudinal fasciculus	Reduced FA	Liao et al. [60]
		Increased AD	Koshiyama et al. [21]		Arcuate fasciculus	Reduced FA	Jiang et al. [83]
	Superior longitudinal fasciculus	Reduced FA	Dong et al. [30]; Jiang et al. [83]		Corona radiata	Reduced FA	van Velzen et al. [61]
		Reduced AD	Lan et al. [82]		Fornix	Reduced FA	van Velzen et al. [61]
	Fornix	Increased MD	Koshiyama et al. [21]		Inferior fronto-occipital fasciculus	Reduced FA	Liao et al. [60]; van Velzen et al. [61]
		Reduced RD	Koshiyama et al. [21]	Post-traumatic stress disorder	Cingulum	Reduced FA	Siehl et al. [75]
	Whole brain – white matter	Reduced RD	Koshiyama et al. [21]		Uncinate fasciculus	Increased MD	Koch et al. [77]
	Cingulum	Reduced FA	Dong et al. [30]; Koshiyama et al. [21]		Corpus callosum	Reduced FA	Rinne-Albers et al. [76]; Siehl et al. [75]
	Inferior longitudinal fasciculus	Reduced AD	Lan et al. [82]			Increased MD and RD	Rinne-Albers et al. [76]
	Uncinate fasciculus	Reduced FA	Dong et al. [30]; Foley et al. [70]		Inferior longitudinal fasciculus	Reduced FA and increased RD	Olson et al. [78]
	Inferior fronto-occipital fasciculus	Reduced FA	Dong et al. [30]		Superior longitudinal fasciculus	Reduced FA	Siehl et al. [75]
Anorexia	Corpus callosum	Reduced FA	Monzon et al. [79]	OCD^a^	Corpus callosum	Reduced FA	Koch et al. [84]
	Fornix	Reduced FA and increased MD	Monzon et al. [79]		Internal capsule	Reduced FA	Koch et al. [84]
	Superior longitudinal fasciculus	Reduced FA and increased MD	Monzon et al. [79]		Cingulum	Reduced FA	Koch et al. [84]; Wang et al. [74]

^a^OCD = obsessive–compulsive disorder

## White matter structure abnormalities in selected anxiety disorders and personality disorders

In the last decade, there has been increasing interest in applying diffusion imaging to assess non-schizophrenic and non-affective mental disorders. This chapter will discuss white matter abnormalities in the most-studied anxiety and personality disorders.

One anxiety disorder whose cause is often seen as biological is obsessive–compulsive disorder (OCD). It is characterized by the presence of intrusive thoughts and repetitive compulsions. Studies have found increased diffusion values—associated with decreased white matter integrity—in the dorsal anterior region of the cingulum of people suffering from OCD. Moreover, a decrease in FA in other structures also correlates with the severity of OCD symptomatology [74]. OCD patients may also have decreased FA in the internal capsule and the corpus callosum [84].

Extremely traumatic experiences, such as accidents, warfare, or abuse, can lead to the anxiety disorder known as post-traumatic stress disorder (PTSD). It is a chronic disease affecting the daily functioning of the individual and its severity may be based on changes within the white matter of the brain [75]. In adults who were sexually abused as children, decreased FA and increased MD and RD, which can be interpreted as a decrease in the integrity of white matter fibers, have been observed in the corpus callosum [76]. However, a study on a group of policemen being treated for PTSD found reduced FA in the right uncinate fasciculus. This was also associated with greater anxiety symptoms in those patients [77]. Post-traumatic stress disorder is also linked with decreased FA in the inferior longitudinal fasciculus [78], superior longitudinal fasciculus, and cingulum [75]. Interestingly, changes in white matter, especially the structures connecting the limbic system (the fornix, cingulum, and fronto-occipital pathways), have also been described in patients diagnosed with anorexia [79]. Unfortunately, the results of the research are still ambiguous and it is not known whether the described changes are primary or secondary parts of the process of the disease. These disorders are presented in Table 4.

Recent studies suggest that white matter integrity disorders may underlie some personality disorders. Borderline personality disorder is the most researched personality disorder. This may be related to the fact that it is the personality disorder associated with the largest number of hospitalizations. It is characterized by instability in various areas of mental life such as emotions and self-image, as well as difficulties with social relationships. It is most frequently related to disorders of connections between the areas of the prefrontal cortex and the limbic system, but also some association bundles connecting the prefrontal cortex with the temporal and occipital areas of gray matter. Decreases in FA may occur in the cingulum, corpus callosum (mainly in the genu, body, and splenium), the left inferior longitudinal fasciculus, and the right superior longitudinal fasciculus [85]. Other studies have noted changes in water diffusion in white matter structures, which may indicate microstructure disorders within the corona radiata, fornix, or thalamic radiations. These deviations may be related to the psychopathology of this disorder, impairment of impulse or emotion control, auto-aggressive behavior, and emotional instability [86]. The lower AD, compared to controls, found in the cingulum, inferior longitudinal fasciculus, and inferior fronto-occipital fasciculus may suggest that the underlying cause of this disorder is white matter pathology [87].

Another personality disorder that has been studied with DTI is antisocial personality disorder, which is characterized by a lack of empathy, impulsive behavior, and a tendency towards aggression and manipulation [88]. A certain pattern of altered AD and RD in patients with impulsive behavior in the population of people with antisocial personality disorder has been detected in the corpus callosum, the posterior part of the corona radiata, thalamic radiations, right superior longitudinal fasciculus, and left inferior longitudinal fasciculus [83]. Another study noted reduced FA values in the right uncinate fasciculus, which connects areas of the amygdala and prefrontal cortex—areas (especially on the right side) which are associated with the psychopathology of psychopathy (pathological lying, superficial charm, and a tendency to manipulate) [89]. Table 5 presents disturbances in diffusion parameters in various fiber bundles in personality disorders.

**Table 5:** Changes diffusion parameters in the brain in borderline and antisocial personality disorders [83, 85–87, 89]

	Bundles	Changes in diffusion parameters	Authors		Bundles	Changes in diffusion parameters	Authors
Borderline personality disorder	Corpus callosum	Reduced FA	Grottaroli et al. [86]; Vandekerckhove et al. [85]	Borderline personality disorder	Superior longitudinal fasciculus	Reduced FA	Grottaroli et al. [86]; Vandekerckhove et al. [85]
		Increased MD	Vandekerckhove et al. [85]		Uncinate fasciculus	Reduced FA	Grottaroli et al. [86]
	Fornix	Reduced FA	Grottaroli et al. [86]	Antisocial personality disorder	Corpus callosum	Reduced AD	Jiang et al. [83]
	Corona radiata	Reduced FA	Grottaroli et al. [86]		Fornix	Reduced FA and AD	Jiang et al. [83]
	Cingulum	Reduced FA	Grottaroli et al. [86]		Corona radiata	Reduced FA and AD, increased RD	Jiang et al. [83]
		Reduced AD	Ninomiya et al. 2018		Internal capsule	Reduced FA and AD	Jiang et al. [83]
	Inferior longitudinal fasciculus	Reduced FA	Grottaroli et al. [86]; Vandekerckhove et al. [85]		Inferior longitudinal fasciculus	Increased RD	Jiang et al. [83]
		Reduced AD	Ninomiya et al. 2018		Uncinate fasciculus	Reduced FA	Jiang et al. [83]; Wolf et al. [89]
	Inferior fronto-occipital fasciculus	Reduced FA	Grottaroli et al. [86]		Inferior fronto-occipital fasciculus	Reduced FA and increased RD	Jiang et al. [83]
		Reduced AD	Ninomiya et al. 2018		Superior longitudinal fasciculus	Reduced FA and AD, increased RD	Jiang et al. [83]

## Summary

We can formulate some basic conclusions based on the above literature analysis regarding the application of magnetic resonance diffusion imaging techniques to psychiatry. First, DTI is widely used in scientific research on psychiatry. The DTI technique is an imaging method that can assess diffusion in tissues and thereby image white matter structures. By assessing various diffusion parameters (including FA, MD, RD, and AD), it is possible to draw conclusions about the properties of white matter fibers. Second, the disconnection hypothesis is one attempt to explain the etiopathogenesis of schizophrenia. It seems that neural communication disorders most often occur between the prefrontal cortex and the cortex of the temporal lobe, limbic system, and occipital lobe. A number of reports discussed in this work also indicate changes in diffusion values in nerve fibers. These changes are often interpreted as diminished integrity in white matter.

Some studies suggest that the occurrence of deficit syndrome is associated with impaired communication between areas of the brain; however, there are currently not enough observations to draw hard conclusions on the matter. Third, white matter connectivity aberrations may underlie affective disorders. Changes in connections between the frontal cortex and the limbic system (i.e., the centers responsible for controlling behavior and regulating emotions) may be associated with the development of depression and bipolar disorder. White matter disorders are probably also important in the etiopathogenesis of anxiety and personality disorders. Unfortunately, there is insufficient research in this area.

It seems necessary to conduct future research using this technique. Thanks to studies done on large populations, it can be said with certainty that there are changes in the structure of white matter associated with many diseases and mental disorders. Future research should focus on the relationship between detailed symptomatology and white matter. Accurate morphological assessment of individual bundles in these disorders will also be important. In future research, it will be very important to pay attention to exogenous factors that may affect the structure and functioning of bundles, such as pharmacotherapy, for example. The future use of neuroimaging in psychiatry depends on the ability to transfer results from research on large groups to the individual level of the patient, which will be significant for clinical practice, diagnosis, therapy, and prognosis [1].

In the future, a good understanding of DTI, its capabilities, and its limitations may be necessary for the everyday practice of psychiatry. Someday, this method may be routinely applied during diagnosis, when planning pharmacotherapy, or for assessing a patient’s prognosis. A basic command of this field will also enable practitioners to better comprehend the processes underlying mental illnesses and disorders.

## Abbreviations

AD: Axial diffusivity
BOLD: Blood-oxygen-level-dependent
BP: Bipolar disorder
DTI: Diffusion tensor imaging
FA: Fractional anisotropy
fMRI: Functional magnetic resonance imaging
MD: Mean diffusivity
MDD: Major depressive disorder
MRI: Magnetic resonance imaging
OCD: Obsessive compulsive disorder
PET: Positron emission tomography
PFC: Prefrontal cortex
PTSD: Post-traumatic stress disorder
RD: Radial diffusivity
SLF: Superior longitudinal fasciculus
SPECT: Single-photon emission computed tomography
SSRIs: Selective serotonin reuptake inhibitors
